# Central nervous system ALK-negative anaplastic large cell lymphoma with IRF4/DUSP22 rearrangement

**DOI:** 10.1007/s10014-021-00415-0

**Published:** 2021-11-18

**Authors:** Shino Magaki, Radha Satyadev, Zesheng Chen, Kathryn S. Yung, Harry V. Vinters, Marsha C. Kinney, Jonathan W. Said

**Affiliations:** 1 Section of Neuropathology, Department of Pathology and Laboratory Medicine, UCLA Medical Center and David Geffen School of Medicine, Los Angeles, CA USA; 2 Department of Pathology and Laboratory Medicine, UCLA Medical Center and David Geffen School of Medicine, Los Angeles, USA; 3 Department of Pathology, Kaiser Foundation Hospital, Anaheim, CA USA; 4 Department of Radiology, Kaiser Permanente Irvine Medical Center, Irvine, CA USA; 5 Department of Neurology, UCLA Medical Center and David Geffen School of Medicine, Los Angeles, CA USA; 6 Brain Research Institute, UCLA Medical Center and David Geffen School of Medicine, Los Angeles, CA USA; 7 Division of Hematopathology, Department of Pathology and Laboratory Medicine, University of Texas Health Science Center at San Antonio, San Antonio, TX USA; 8 Present Address: Centre Hospitalier Universitaire Sainte-Justine, Montréal, QC Canada

**Keywords:** Central nervous system, Anaplastic large cell lymphoma, *ALK*, *IRF4/DUSP22*

## Abstract

Anaplastic large cell lymphomas (ALCL) are mature T-cell neoplasms, approximately half of which harbor rearrangements of the *ALK* gene that confer a good prognosis. Recent studies have demonstrated that a significant proportion of ALK-negative ALCLs demonstrate rearrangements of the *IRF4/DUSP22* locus that also are typically associated with a favorable prognosis. ALCL with primary involvement of the central nervous system (CNS) is extremely rare. We report what may be the first case of ALK-negative ALCL with *IRF4/DUSP22* rearrangement involving the brain in a 55-year-old man. Magnetic resonance imaging demonstrated signal abnormalities in the periventricular region, corpus callosum and cingulate gyrus. Biopsy revealed a diffuse parenchymal and angiocentric infiltrate of CD30-positive cells that showed *IRF4/DUSP22* rearrangement by fluorescence in situ hybridization. We also review the clinical and pathologic features of primary CNS ALK-negative ALCLs in the literature and highlight the need for awareness of this entity to optimize appropriate management.

## Introduction

Anaplastic large cell lymphomas (ALCLs) are mature T-cell neoplasms expressing CD30, a lymphocyte activation marker [1–3]. They are heterogeneous genetically and clinically, and show a morphologic spectrum as well as overlap with other T-cell lymphomas, sometimes making diagnosis difficult [4–6]. According to the World Health Organization (WHO), there are four types of ALCLs, systemic ALK-positive ALCL, systemic ALK-negative ALCL, primary cutaneous ALCL, and the provisional entity breast-implant-associated ALCL [3, 4]. Approximately half of systemic ALCLs show translocations involving the anaplastic lymphoma kinase gene *ALK* on 2p23, which is associated with a good prognosis, partly due to ALK-positive ALCL being more common in children and young adults whereas ALK-negative ALCL peaks in middle age [1, 3]. It has recently been shown that of the remaining half of ALK-negative ALCLs, approximately 20–30% show rearrangements of the *DUSP22-IRF4* locus on 6p25.3, 2–8% have rearrangement of *TP63* on 3q28 and the remainder lack all three alterations (i.e., “triple negative”), with these alterations appearing to be mutually exclusive in most cases [1, 7, 8]. The *DUSP22* gene is immediately telomeric (40 kb) to *IRF4* at the 6p25.3 locus, and *IRF4/DUSP22* break-apart FISH probes flanking these genes detect rearrangements involving both *IRF4* and *DUSP22* but cannot distinguish between the two [9, 10]. Rearrangement of this locus is often called *DUSP22* rearrangement as it is associated with decreased expression of dual-specificity phosphatase-22, which regulates mitogen-activated protein kinase signaling, but not *IRF4* [1, 9, 10]. *DUSP22* rearranged ALCLs typically have a favorable prognosis, similar to *ALK* rearrangements with 5-year overall survival (OS) of 80–90% [1, 4, 11]. Alterations in *TP63* are associated with a poor prognosis and lack of any of these rearrangements with an intermediate prognosis, with 5-year OS of 0–17% and 33–42%, respectively [1, 11]. *MYC* rearrangement has been rarely reported in ALK-negative ALCL with 2 patients recently described by Khanlari et al. both of whom had aggressive disease although one demonstrated a concurrent *DUSP22* rearrangement and had longer survival compared to the patient without the alteration [12].

The majority of primary central nervous system lymphomas (PCNSL), non-Hodgkin lymphomas restricted to the CNS, including brain, meninges, spinal cord or eye at presentation, are of the diffuse large B-cell type with T-cell lymphomas constituting less than 5% [13]. ALCLs involving the CNS are very rare, have been ALK-positive, ALK-negative or with unknown ALK status, and tend to exhibit aggressive behavior [5, 13, 14]. Only 15 cases of primary CNS ALK-negative ALCLs have been reported [5, 13, 15–24]. We describe what may be the first reported case of primary CNS ALK-negative ALCL with *DUSP22* rearrangement and review the clinical and pathologic characteristics of CNS ALK-negative ALCLs in the literature.

## Clinical summary and pathologic findings

**Fig. 1:**
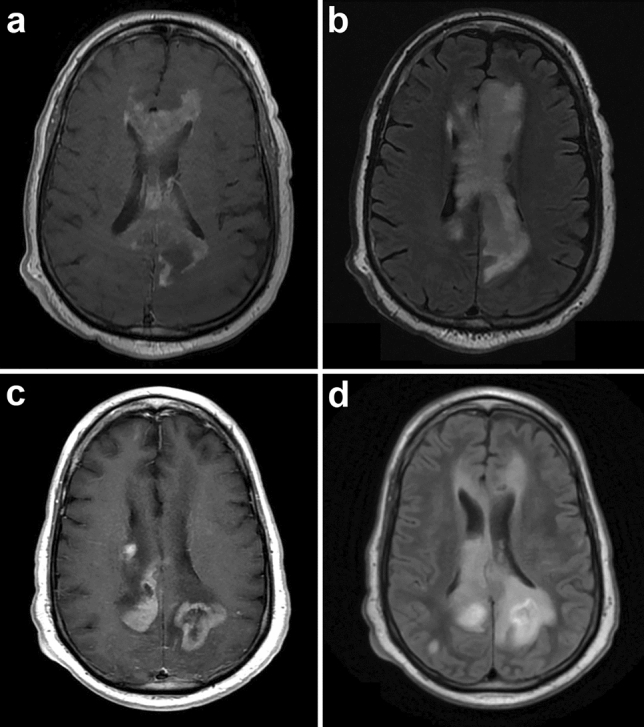
Initial axial, contrast-enhanced T1-weighted, magnetic resonance image demonstrating enlargement and amorphous enhancement involving the cingulate gyrus and corpus callosum, extending across the midline (**a**). There was also confluent periventricular white matter T2/FLAIR hyperintensity (**b**). Repeat imaging 3 months later showed multiple new contrast-enhancing masses (**c**) and progression of T2/FLAIR hyperintensity (**d**)

A 55-year-old man with history of hypertension, dyslipidemia, prediabetes, asthma, microcytic anemia likely due to thalassemia, and cardiomyopathy presented with lightheadedness for 2 weeks followed by problems with balance resulting in several falls as well as irritability, memory problems and confusion for 1.5 weeks. He also had difficulty using his right hand. Magnetic resonance imaging (MRI) of the brain showed enlargement and amorphous enhancement involving the left cingulate gyrus, which extended into the left temporal lobe, hippocampus and corpus callosum with extension across the midline (Fig. 1). Additionally, confluent periventricular white matter T2/FLAIR hyperintensity was seen. Cerebrospinal fluid (CSF) cytology performed twice was negative for malignant cells. CSF flow cytometry showed mostly CD5 positive T cells and was interpreted as negative for a lymphoproliferative disorder. HIV testing was negative. Computed tomography (CT) of the neck, chest, abdomen, and pelvis at presentation and repeated approximately one month later showed no convincing evidence of malignancy. Although a PET scan had not been done, CT of the chest, abdomen and pelvis was repeated a third time and was again negative. He also did not have B symptoms of fever, night sweats and weight loss.

**Fig. 2:**
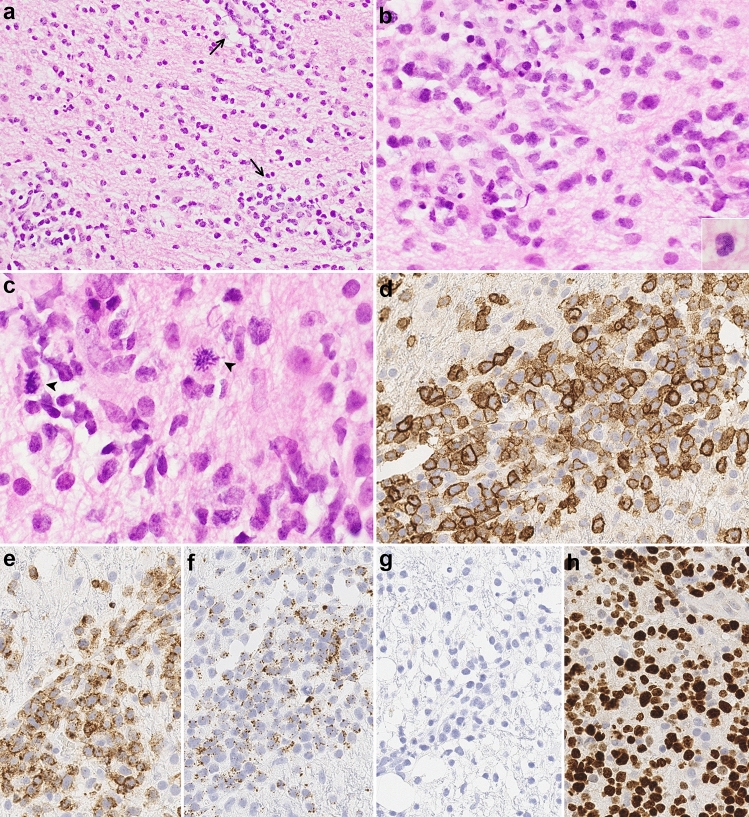
Biopsy of the corpus callosum lesion showing large pleomorphic cells, admixed with medium to small lymphocytes, diffusely infiltrating brain parenchyma with perivascular accentuation (arrows) (**a**, 200 ×). The cells have irregular nuclear contours (**b**, 400 ×) with scattered hallmark cells (inset 600 ×); mitotic figures are easily seen (arrowheads) (**c**, 600 ×). On immunohistochemistry, the cells are positive for CD30, with many showing strong membranous and Golgi staining (**d**, 400 ×); large cells negative for CD30 are likely reactive astrocytes, endothelial cells and/or histiocytes. The tumor cells are also positive for CD2 (**e**, 400 ×) and granzyme B (**f**, 400 ×). ALK-1 is negative (**g**, 400 ×). The MIB-1 labeling index is over 90% (**h**, 400 ×)

**Fig. 3:**
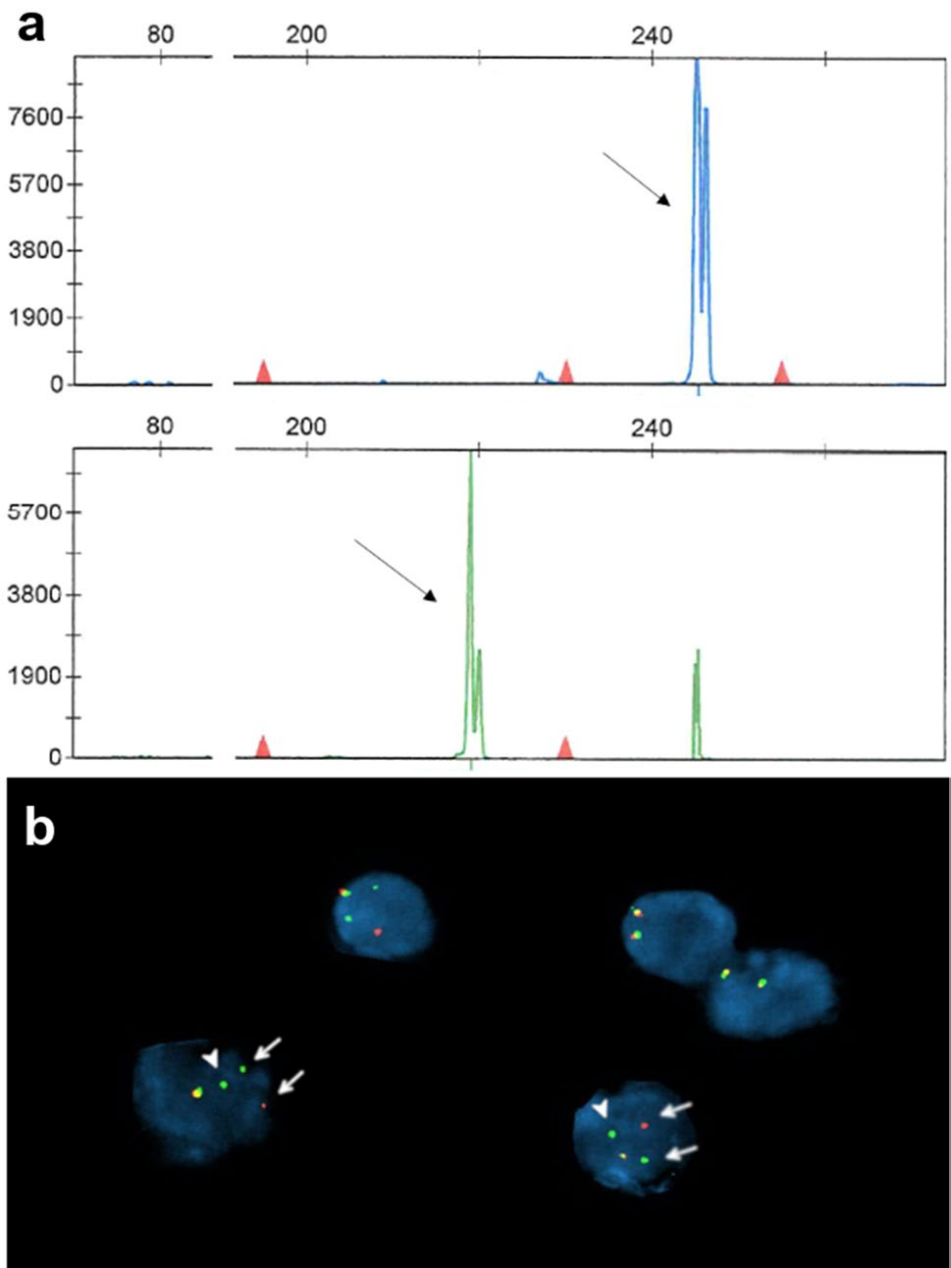
*TCRG* clonal product (arrows at 245 bp and 218 bp) with two of the primer sets using the BIOMED-2 protocol (**a**). Fluorescence in situ hybridization demonstrates *IRF4/DUSP22* rearrangement (arrows) with an extra copy of *3′IRF4* in the large cells (arrowheads) (**b**)

The patient underwent a left frontal brain biopsy that showed atypical lymphoid cells in a background of reactive brain tissue with gliosis and lymphohistiocytic infiltrate. The large cells were positive for CD30 and predominantly negative for CD3. Although features were suspicious for a lymphoproliferative disorder, the sparsity of the atypical cells made definitive diagnosis challenging and also limited molecular testing. He was placed on corticosteroids, but subsequent MRI showed multifocal heterogeneous enhancement with progression of abnormal signals in some regions, with decreasing signals in others. A repeat brain biopsy of the corpus callosum and cingulate gyrus was performed 1.5 months later and showed an infiltrate of many large cells admixed with medium-sized and small lymphocytes in a perivascular and diffuse parenchymal distribution (Fig. 2). The large cells had irregular nuclear contours with small to indistinct nucleoli and moderate amounts of cytoplasm. Scattered hallmark cells with kidney-shaped nuclei were seen, but there were no “doughnut” cells with central nuclear pseudoinclusions or multinucleated cells with “wreath-like” nuclei. Mitoses were easily identified, but no necrosis was seen. The background brain tissue showed gliosis, highlighted by immunohistochemistry for GFAP. The large cells were positive for CD2, CD4, CD25, CD30, and expressed the alpha–beta T-cell receptor (TCR) protein as detected by the antibody βF1 and had negative staining for TCR delta. There was decreased expression of CD3, CD5 and CD7. The neoplastic cells were also positive for granzyme B with a subset positive for TIA-1 and negative for CD8, ALK-1, EMA, clusterin, and EBV EBER. The MIB-1 labeling index was over 90% in the large cells. Less than 5% of the cells were B cells (CD20 +, PAX5 +), and a small population of polytypic plasma cells was present. PCR testing for TCR gene rearrangements showed a clonal gene arrangement at the *TRG* locus (Fig. 3a). Fluorescence in situ hybridization (FISH) studies were performed with the Vysis FISH *ALK* break-apart probes against the 5’ to 3’ region of the *ALK* gene (2p23) and the Leica FISH *IRF4/DUSP22* break-apart probes against the 5′ to 3′ region of the *IRF4* and *DUSP22* genes (6p25.3) which demonstrated an *IRF4/DUSP22* gene rearrangement with gain of 3′IRF4 in over 90% of nuclei assessed (Fig. 3b). No *ALK* gene rearrangement was detected.

Unfortunately, the patient developed seizures and aspiration pneumonia and had to be placed on mechanical ventilation. Repeat brain MRI showed multifocal enhancing masses increased in number and size (Fig. 1c, d). He was placed on multiple anticonvulsant medications and corticosteroids, but due to multiorgan injury could not be given chemotherapy or radiation. He was referred to hospice care and died 2.5 months after the initial surgery.

## Discussion

PCNSLs are rare, accounting for 1–2% of non-Hodgkin lymphomas and 2% of all primary brain tumors [13, 14, 25]. The majority are B-cell lymphomas, with DLBCL comprising over 90% [13, 26, 27]. T-cell lymphomas account for approximately 2–4% of all primary CNS lymphomas in the West [28, 29] with a higher incidence in Asia, ranging from 2 to 17% [30–32]. Primary CNS T-cell lymphomas (PCNSTL) include peripheral T-cell lymphoma, not otherwise specified, the most common subtype, ALCL, and extranodal NK/T-cell lymphoma [5, 26, 32, 33]. In a series of 45 PCNSTLs from multiple institutions and countries, Shenkier et al. reported a wide age range of presentation from 3 to 84 years with median age of 60 years and male predominance, similar to PCNSLs overall [27, 34]. In a series of primary CNS ALCLs, including ALK-positive, ALK-negative and ALK status unknown, George et al. examined 9 cases, 5 previously reported, and reviewed an additional 4 cases in the literature [5]. All cases evaluated had supratentorial lesions with 2 additionally involving the infratentorial compartment and one also involving the spinal cord [5]. Nine of the 13 cases showed dural/leptomeningeal involvement including 2 cases with sole involvement of the dura, one ALK positive and the other with ALK status unknown [5].

Of the 15 patients with primary CNS ALK-negative ALCL reported in the literature and the current case (Table 1), the median age was 62 (range 22–82). Except for one young female patient who was 22 years old, all patients were above 45 years of age, in contrast to CNS ALK-positive ALCLs, which have been reported in children, similar to systemic ALK-positive ALCL [5, 35]. There was a nearly equal gender distribution with 8 females and 7 males. Nine of 13 patients had a normal immune system. Symptoms spanned a few weeks to several months and ranged from focal neurologic deficits, seizures, and headaches to symptoms of dementia. This highlights the importance of considering CNS lymphoma in the differential diagnosis of atypical dementia symptoms as it is one of the most common diagnoses on biopsies done for neurologic decline of unknown etiology [36]. Nearly all cases involved the supratentorial compartment as solitary or multifocal lesions except in one patient (case 14) who showed leptomeningeal involvement of the posterior fossa and spinal cord [16]. Seven subjects had solitary lesions, whereas 8 had multifocal or diffuse involvement on MRI. Six patients showed leptomeningeal involvement on imaging or histologic examination, some centered in the dura. Seven of 8 patients tested, including the present case, were positive for TCR gene rearrangements with one case resulting in no amplification products (Table 2).

**Table 1:** Clinical and imaging characteristics of ALK-negative ALCL in the central nervous system^a^

Case	Reference	Year	Age	Sex	Immune status	Clinical presentation	Focality	Location/site	Dural, leptomeningeal involvement^b^
1	Paulus et al. George et al.	1994 2003	63	M	Normal	2 weeks of left arm paresis, Jacksonian epileptic seizures	Multifocal/diffuse	3 right fronto-parietal masses (dura and brain)	Yes
2	Nuckols et al. George et al.	1999	66	F	Systemic lupus erythematosus, chronic renal failure, thymoma	NA	Single	Right temporal	No
		2003							
3^c^	Chuang et al. George et al.	2001	46	F	Normal	2 weeks of headache, right sided weakness and left eye blurred vision	Single	Left parieto-occipital (dura and brain)	Yes
		2003							
4	George et al.	2003	22	F	Normal	NA	Multifocal/diffuse	Cerebellum, 4 additional infra- and supratentorial sites	No
5	George et al.	2003	50	F	Normal	NA	Multifocal/diffuse	Right parietal, 2 additional supratentorial and dural sites	Yes
6	Gonzales et al.	2003	82	F	NA	3 months of lower cranial nerve signs	Single	Tentorium cerebelli	Yes
7	Tajima et al.	2003	52	F	Essential thrombocythemia on hydroxyurea	15 months of gradual worsening right hemiparesis	Multifocal/diffuse	Widely distributed lesions in bilateral frontal lobes	Not reported
8	Rowsell et al.	2004	46	M	HIV, Crohn disease	3 weeks of progressive ataxia, inability to ambulate	Single	Right occipital	Not reported
9	Kodama et al.	2009	79	M	Normal	2 weeks of dementia-like symptoms (sensory dominant aphasia, dressing ataxia, agraphia, acalculia)	Single	Left parieto-occipital	Not reported
10	Colen et al.	2010	65	M	Normal	Progressive headaches and blurry vision, left eye proptosis, history of atypical meningioma status post near total resection and radiation 2.5 years prior to presentation	Single	Floor of left middle cranial fossa	Yes
11	Sugino et al.	2013	75	M	Normal	Insidious onset of memory loss followed 2 months later by rapidly progressing dementia	Multifocal/diffuse	White matter of bilateral cerebral hemispheres	No
12	Menon et al.	2015	61	F	NA	Right superior extremity weakness, paresthesia, mild paralysis	Multifocal/diffuse	Diffuse enhancement	No
13	Menon et al.	2015	62	F	Multiple sclerosis	3 months of left lower extremity weakness	Single	Right frontal	No
14	Lannon et al.	2020	63	M	Normal	Insidious bilateral leg weakness for 3 months, left facial numbness, weakness, dysarthric speech, left eye blurred vision, headaches, progressive fatigue, weight loss, multiple cranial nerve involvement, partial sensory level at C5	Multifocal/diffuse	Extensive multifocal leptomeningeal enhancement of spinal cord, brainstem and cerebellum	Yes
15	Present case	2020	55	M	Normal	3.5 weeks of lightheadedness, balance problems with falls, difficulty using right hand	Multifocal/diffuse	Left cingulate, temporal lobe, hippocampus and corpus callosum	No

*NA* not available

^a^All CD30 positive; cases in which ALK status was not available were excluded

^b^On imaging or histologic examination

^c^Patient 3 had undergone tumor excision at a different hospital 2 months prior to presentation at authors’ institution with diagnosis of possible B-cell lymphoma and no follow-up treatment

**Table 2:** Pathologic characteristics and management of ALK-negative ALCL in the central nervous system^a^

Case	Immunophenotype (all CD30 +, ALK −)	Necrosis	Treatment	Outcome	T-cell receptor (TCR) gene rearrangement	CSF cytology	CSF flow cytometry
1	CD3 + (> 50%), CD20 − CD45 −, CD45RO +, EMA + (50%), HLA-DR +	Yes	Radiation	Died 11 weeks after symptom onset	Positive (TRB)	Negative	NA
2	CD3 +, CD15 −, CD20 −	Yes	Supportive	Died 4 days after surgery	NA	NA	NA
3	CD3 −, CD15 −, CD20 −, CD43 +, CD45RO −, CD79a −, TIA-1 +, granzyme B +, EBV EBER +, EMA −	Yes	Radiation	No evidence of disease at 25 months	Positive (TRG)	NA	NA
4	CD3 +, CD8 +, EBV EBER −	Yes	Supportive	Died 11 days after surgery	NA	NA	NA
5	Negative for T-cell and B-cell markers	Yes	Radiation	Died 2 months after surgery	NA	NA	NA
6	CD3 −	Not reported	Supportive	Died 6 weeks after surgery	NA	NA	NA
7	CD3 −, CD20 −, CD45 −, CD56 −, EMA −	Yes	Methotrexate, radiation	NA	NA	Reactive	NA
8	CD2 +, CD3 −, CD5 −, CD20 −, CD43 +, CD45 +, CD79a −, bcl-2 +, EMA +	Not reported	Radiation	Died 2 months after diagnosis	Positive (TRG)	NA	NA
9	CD3 +, CD5 +, CD15 −, CD20 −, CD45RO +, CD56 −, CD79a +, granzyme B +, EMA +, EBV EBER −	Yes	Supportive	Died 4 months after initial surgery	Positive (TRB)	NA	NA
10^b^	CD3 +, CD15 −, EMA −, TIA-1 −	Not reported	High dose methotrexate, CHOP	Clinically stable on CHOP as of report	NA	NA	NA
11	CD3 −, CD15 −, CD20 −, CD43 +, CD45RO −, CD79a −, Bcl-6 −, EMA −	No	Methylprednisolone, radiation	Died 8 months after symptom onset	NA	Many lymphoid cells without atypia	NA
12	CD2 +, CD3 +, CD4 −, CD5 +, CD7 + (focal), CD8 + (weak), βF1 +, TCRγ −, TIA-1 + (focal), granzyme B +	No	Dexamethasone	Died of disease at 1 month	No amplification products	NA	NA
13	CD3 + (weak), CD5 −, CD56 −, TIA-1 −, EBV −	Yes	NA	NA	Positive (TRG)	NA	NA
14	CD2 +, CD3 +, CD8 +, CD20 −, CD43 +, CD79a −, granzyme B +, MUM-1 +, EBV −	Not reported	Dexamethasone, methotrexate, cytarabine, thiotepa	Well 18 months after diagnosis	Positive	Negative	Negative
15	CD2 +, CD3 + (decreased), CD4 +, CD5 + (decreased), CD7 + (decreased), CD8 −, CD25 +, CD56 −, βF1 +, TCRγ −, granzyme B +, TIA-1 + (subset), EMA −, clusterin −, EBV EBER −	No	Dexamethasone	Died 2.5 months after initial surgery	Positive (TRG)	Negative	Negative

*NA* not available; *CHOP* cyclophosphamide, doxorubicin, vincristine, and prednisone

^a^All CD30 positive; cases in which ALK status was not available were excluded

^b^Patient 10 had synchronous ALCL and recurrent atypical meningioma

Systemic ALK-positive ALCLs demonstrate several histologic patterns with the “common” pattern of sheet-like growth of hallmark cells with kidney-shaped nuclei, the most frequent pattern among all types of ALCLs [37]. Other variants include the lymphohistiocytic pattern with admixed abundant reactive histiocytes, Hodgkin-like pattern resembling nodular sclerosis classic Hodgkin lymphoma (although most cases previously diagnosed with this pattern are likely classical Hodgkin lymphomas and not ALCLs), and small-cell pattern [3, 4, 37, 38]. Case 3 was reported to be positive for EBV EBER by in situ hybridization and showed abundant histiocytes and eosinophils, suggestive of the Hodgkin-like pattern [5, 19]. As ALCLs are consistently negative for EBV according to the WHO, case 3 may not fit into the category of ALK-negative ALCL with current criteria and may represent rare intracerebral Hodgkin lymphoma [3, 37, 39, 40]. ALK-negative ALCLs show morphologic patterns similar to ALK-positive ALCLs, and a small-cell pattern is not recognized due to overlap with other peripheral T-cell lymphomas [4].

King et al. in a series of systemic ALK-negative ALCLs with *DUSP22* rearrangement, have shown that the majority show the common pattern with some cases having slightly smaller cells compared to other genetic subtypes. ALCLs with *DUSP22* rearrangement were more likely to show “doughnut” cell morphology with central nuclear pseudoinclusions and less likely to show large pleomorphic and/or multinucleated cells which have been reported to be more common in ALK-negative compared to ALK-positive ALCL but less often seen in ALCL with *DUSP22* and *TP63* rearrangements [1, 4]. The current case did not demonstrate “doughnut” cells or multinucleated cells. Of the prior reported cases of primary CNS ALK-negative ALCL, no “doughnut” cells were reported, but 5 cases described few to frequent multinucleated cells. Although necrosis was not seen in our case, necrosis is a common feature in PCNSTLs [13] and CNS ALK-negative ALCLs (8 of 15). Perivascular cuffing is also frequently, but not always, seen in primary CNS lymphomas of both B-cell and T-cell lineage, and T-cell lymphomas can be challenging to diagnose as the brain often shows florid reactive changes with gliosis and histiocytic infiltrate, obscuring diffuse parenchymal involvement [13, 14]. On immunohistochemistry, EMA and clusterin are more often negative, and CD2 and CD3 more frequently positive in all genetic subtypes of ALK-negative ALCL compared to ALK-positive ALCL [1, 7]. Similarly, the present case showed absent expression of EMA and clusterin with positivity for CD2 but with decreased expression of CD3. Of the CNS ALK-negative ALCL cases, 9 of 15 had some degree of CD3 immunopositivity, while only 3 of 8 cases on which EMA was performed showed EMA expression (Table 2). While immunohistochemistry for cytotoxic markers is not specific for ALCL subtype, only 5–10% of *DUSP22* rearranged cases have been shown to express the cytotoxic markers granzyme B and TIA-1 [1]. The current case was one of the 5 cases positive for TIA-1 and/or granzyme B out of 7 CNS ALK-negative ALCLs on which they were performed. A unique finding in our case is the presence of an extra copy of *3*′*IRF4* in addition to the *IRF4/DUSP22* rearrangement. In a study of 182 patients with cutaneous T-cell lymphoproliferative disorders, Wada et al. found 9 of 45 cutaneous ALCLs and 1 of 32 cases of lymphomatoid papulosis to have an *IRF4/DUSP22* translocation. Other *IRF4* alterations, most commonly extra copies of *IRF4*, were seen in a wide variety of T-cell lymphoproliferative disorders, including cutaneous ALCL, and were mutually exclusive with cases harboring *IRF4/DUSP22* translocations [10]. The effects of additional cytogenetic alterations on prognosis are unknown; rare cases of *DUSP22* rearranged ALCLs demonstrating other alterations, one with concurrent *TP63* rearrangement and another with concurrent *MYC* rearrangement, showed complete remission after chemotherapy although the patient with *DUSP22* and *MYC* rearrangements died 53 months after diagnosis from an unknown cause [8, 12].

There is no consensus on the optimal therapy for PCNSL, but treatment most often consists of high-dose methotrexate as part of multiagent chemotherapy, with or without radiation [41]. Most aggressive systemic lymphomas, including ALCL, are treated with an anthracycline containing chemotherapy regimen, such as cyclophosphamide, doxorubicin, vincristine, and prednisone (CHOP) [1, 42]; however, these regimens are ineffective in PCNSL in part due to insufficient penetration of the blood–brain barrier (BBB) [41]. Brentuximab vedotin, an antibody–drug conjugate comprised of anti-CD30 monoclonal antibody conjugated to the anti-microtubule agent monomethyl auristatin E, has shown efficacy for relapsed/refractory ALCL but is also not thought to cross the BBB [1, 42, 43].

PCNSLs tend to have a worse prognosis compared to extra-CNS lymphomas [41]. The 5-year OS of PCNSTLs is approximately 30%, similar to that of PCNSL, DLBCL type [44]. ALK-positive ALCL has a better prognosis compared to ALK-negative ALCL also in the CNS, but both appear to have a worse prognosis compared to extra-CNS ALCLs, although studies are limited by the rarity of the disease [5, 35]. Among the CNS ALK-negative ALCLs, 10 of 13 patients with available outcome data died 4 days to 6 months after surgery. Moreover, ALCLs with *DUSP22* rearrangement have usually been associated with a good prognosis, but Hapgood et al. reported a 5-year OS of approximately 40% in ALCLs with *DUSP22* rearrangement in their series, although their 5-year OS of ALK-positive ALCLs were also lower at 69%, which may be due to the difference in populations studied [7].

In summary, ALK-negative ALCL with *DUSP22* rearrangement can also present primarily in the brain and in our case had a poor outcome. T-cell lymphomas are rare in the CNS and, thus, require a high level of suspicion for the appropriate diagnosis to be made. Although not entirely specific, TCR gene rearrangements may be helpful. ALCLs are clinically and genetically heterogeneous, and this case emphasizes the importance of assessing for recurrent fusions involving *ALK*, *DUSP22* and *TP63,* which have a significant impact on prognosis and management.
